# Hearing Loss in Neurological Disorders

**DOI:** 10.3389/fcell.2021.716300

**Published:** 2021-08-11

**Authors:** Siyu Li, Cheng Cheng, Ling Lu, Xiaofeng Ma, Xiaoli Zhang, Ao Li, Jie Chen, Xiaoyun Qian, Xia Gao

**Affiliations:** ^1^Department of Otolaryngology Head and Neck Surgery, Affiliated Drum Tower Hospital of Nanjing University Medical School, Jiangsu Provincial Key Medical Discipline (Laboratory), Nanjing, China; ^2^Research Institute of Otolaryngology, Nanjing, China

**Keywords:** hearing loss, neurodegenerative diseases, autism spectrum disorder, pathological mechanisms, molecular mechanisms

## Abstract

Sensorineural hearing loss (SNHL) affects approximately 466 million people worldwide, which is projected to reach 900 million by 2050. Its histological characteristics are lesions in cochlear hair cells, supporting cells, and auditory nerve endings. Neurological disorders cover a wide range of diseases affecting the nervous system, including Alzheimer’s disease (AD), Parkinson’s disease (PD), Huntington’s disease (HD), autism spectrum disorder (ASD), etc. Many studies have revealed that neurological disorders manifest with hearing loss, in addition to typical nervous symptoms. The prevalence, manifestations, and neuropathological mechanisms underlying vary among different diseases. In this review, we discuss the relevant literature, from clinical trials to research mice models, to provide an overview of auditory dysfunctions in the most common neurological disorders, particularly those associated with hearing loss, and to explain their underlying pathological and molecular mechanisms.

## Introduction

Hearing loss is defined by an average pure-tone threshold detection exceeding 20 dB, affecting approximately 466 million people worldwide. According to the value of pure tone thresholds, it can be classified as mild (20–35 dB), moderate (35–50 dB), moderately severe (50–65 dB), severe (65–80 dB), profound (80–95 dB), and total (≥95 dB) hearing loss. Lesions in the cochlea, auditory nerve, and central auditory pathway induce sensorineural hearing loss (SNHL); nearly a third of the population over the age of 65 is suffering from it^1^. Histological characteristics of age-related hearing loss include degenerative pathology in cochlear hair cells, supporting cells, and auditory nerve endings, resulting in irreversible damage to the sensory epithelium of the cochlea (He et al., 2020; Keithley, 2020; Wu et al., 2020b). Sound is collected and conducted by the external and middle ear, then transformed into electrical signals by cochlear mechanosensory cells: the inner and outer hair cells (OHCs) (Dallos, 1986). OHCs function to enhance sound frequency selectivity and mechanical amplification, and inner hair cells (IHCs) are responsible for subsequent sound detection and transmission. Hair cells are sensitive to aging, acoustic trauma, ototoxic drugs (Fu et al., 2021b), and environmental or genetic influences (Wang et al., 2017; Qian et al., 2020; Fu et al., 2021a; Lv et al., 2021). As damages to either type of hair cells can result in permanent SNHL, many studies have focused on biological treatments for hearing restoration, including gene therapy, hair cell regeneration, etc. (Liu et al., 2016; Li et al., 2018b; Chen et al., 2021; He et al., 2021). These electrical signals are then transduced to the auditory cortex by spiral ganglion neurons (SGNs). SGNs are located in the Rosenthal’s canal of the cochlea and work as the primary sensory neurons to connect the peripheral and central auditory systems, which are susceptible to aging and ototoxic drugs. Hence, preventing the degeneration of SGNs carries critical implications for improving the restoration of hearing (Appler and Goodrich, 2011; Coate and Kelley, 2013; Sun et al., 2016; Liu et al., 2019, 2021; Guo et al., 2021). The pulses ascend into the cochlear nuclei, superior olivary complex, and inferior colliculus for the perception of time and intensity, then target toward the medial geniculate body, and finally, the auditory information is integrated into and further processed by the auditory cortex (Grothe et al., 2010; Profant et al., 2015; Wu et al., 2015). The ascending and reversed descending pathways (originating from the cerebral cortex to the cochlea) form the complete auditory circuitry. Pathology in any portion of the auditory circuitry will lead to auditory dysfunctions, including hearing impairments and central auditory processing disorder, which can be addressed through pure tone audiometry (PTA) and speech tests (such as speech discrimination and speech-in-noise tests). The effects of hearing loss are widespread and profound, resulting in social isolation, psychological illness. And hearing loss is reported to be closely associated with cognitive decline and dementia independently in the elderly population (Lin et al., 2011a, 2013; Jafari et al., 2019).

Neurological disorders include a broad range of diseases that affect the nervous system, of which neurodegenerative diseases and neurodevelopmental disorders have been widely discussed. In the elderly, neurodegenerative diseases are common causes of morbidity and cognitive impairment (Kritsilis et al., 2018; Hou et al., 2019). The progression of these diseases is characterized by the diffusion of protein aggregates, which correlates with clinical severity (Ross and Poirier, 2004; Herrero and Morelli, 2017; Davis et al., 2018). Autism spectrum disorder (ASD) is a neurodevelopmental disorder characterized by social isolation, stereotypical behaviors, and interests. Genetic and environmental risk factors jointly account for phenotypic variations in ASD (Johnson et al., 2007; Lai et al., 2014a). Recent studies have reported that patients suffering from these neurological disorders are accompanied by hearing impairments and other auditory dysfunctions, especially in Alzheimer’s disease (AD), Parkinson’s disease (PD), Huntington’s disease (HD), and ASD. Meanwhile, many mechanisms may account for the complex interplay, including neuropathological changes in the central and peripheral auditory system, social isolation caused by hearing decline, or other potential molecular mechanisms (Fortunato et al., 2016; Shen et al., 2018). It remains unclear whether auditory dysfunction is intrinsic or secondary to these diseases. Here, we discuss the relevant clinical literature to review the most common neurological disorders, particularly those associated with hearing loss, and explain their underlying pathological and molecular mechanisms.

## Alzheimer’s Disease

Alzheimer’s disease is a progressive neurodegenerative disorder and the most common form of dementia. One in 10 people aged over 65 years is affected by AD, and the incidence increases with age (Soria Lopez et al., 2019; Alzheimer’s Association, 2020). Late-onset AD (LOAD) is the onset of AD later than 65 years of age, accounting for approximately 94% of all cases. Symptomatic AD exhibits insidious impairments in learning and memory at the initial stage, and then progresses toward impairments in cognition and executive function at the later stage (Long and Holtzman, 2019). AD patients are usually present with deficient perceptual and semantic processing of sounds (Perez et al., 2009; Benarroch, 2010; Ruan et al., 2012; Attems et al., 2014; Albers et al., 2015; van Wijngaarden et al., 2017). Since the 1980s, the association between hearing impairments and AD has been discussed. Evidence has shown that cognitive impairment is often accompanied by hearing loss, and in turn, hearing loss increases the incidence of cognitive decline and AD (Gallacher et al., 2012; Hung et al., 2015; Panza et al., 2015; Fortunato et al., 2016; Ford et al., 2018). Ford et al. (2018) estimated that midlife hearing loss might account for 9.1% of dementia cases globally. Lin et al. (2011a) demonstrated that for every 10 dB increase above the pure tone threshold of 25 dB, the risk of dementia increased by approximately 20%, with risk ratios for mild, moderate, and severe hearing loss of 1.89, 3.00, and 4.94, respectively. Taljaard et al. (2016) conducted a meta-analysis illustrating that hearing impairments coexisted with more inferior cognitive ability in older individuals, and receiving hearing interventions improved cognitive outcomes.

**FIGURE 1:**
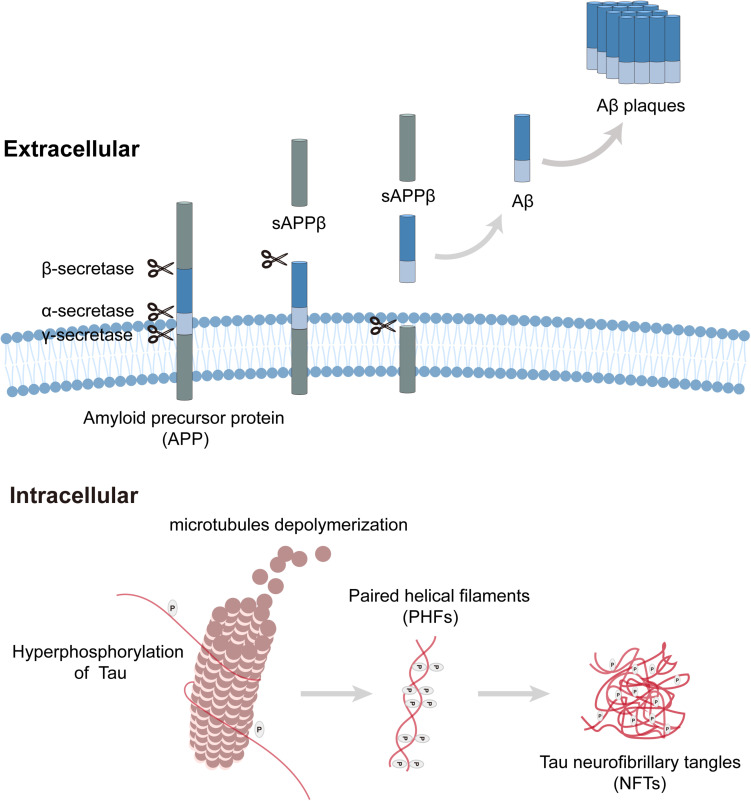
Amyloid plaque formation extracellular and tau pathology intracellular. Amyloid precursor protein (APP) is a transmembrane protein that can be cleaved by three kinds of secretases. In the process of amyloid plaque formation, APP is cleaved by β-secretase and γ-secretase sequentially, then amyloid-β peptides release to extracellular and clump together to form deposits (Aβ plaques). Tau plays a critical role in microtubule assembly and stabilization, hyperphosphorylation of tau leads to microtubules depolymerization, and paired helical filaments (PHF) aggregate to form tau neurofibrillary tangles (NFTs).

Neuropathological changes in the auditory system of AD have been widely explored, and typical AD pathological changes have been observed in auditory pathways (Uhlmann et al., 1986). Extracellular amyloid-β (Aβ) peptide aggregation and intracellular neurofibrillary tangles (NFTs) are the neuropathological hallmarks of AD (Figure 1; Long and Holtzman, 2019). In the amyloidogenic pathway, amyloid precursor proteins (APP) are membrane proteins that are sequentially cleaved by β-secretase and γ-secretase, resulting in the release of extracellular amyloid-β peptides, where they clump together to form deposits (Aβ plaques) and initiate a cascade of pathogenic processes and neurodegeneration. Tau protein plays a critical role in the development of neurons, and its hyperphosphorylation leads to the production of NFTs. Aβ peptides and NFTs coalesce to induce cellular dysfunctions (inflammation, oxidative stress, etc.), synaptic loss, and neurodegeneration (Guo et al., 2017; Makin, 2018). Genetically, AD is classified into familial (FAD) and sporadic cases. FAD accounts for 5% of AD cases and has an autosomal dominant inheritance pattern. Mutations in *APP*, *PSEN1* (Presenilin 1), and *PSEN2* (Presenilin 2) are responsible for the occurrence of FAD, and it was reported that mutations in these genes alter APP processing, induce Aβ formation, and then initiate tau pathology. In contrast, more than 90% of AD patients appear sporadically, which usually presents with late-onset AD (Piaceri et al., 2013). The only confirmed risk gene for sporadic AD is apolipoprotein E (APOE), which encodes an amino acid lipoprotein that can bind to amyloid precursor proteins. The Epsilon4 allele in APOE is strongly associated with an increased risk of AD in either homozygous or heterozygous states. Over 60% of sporadic cases are unrelated to APOE, suggesting that the interplay of genetic and environmental elements contributes to the occurrence of sporadic AD (Verghese et al., 2011). Similar neuropathology is also observed in sporadic AD without such mutations, indicating that Aβ plaques may be the driving force behind tau pathology, but not the sole one (van der Kant et al., 2020).

In the early stage of AD, brain atrophy occurs in the central auditory cortex and related functional nuclei; senile plaques (SPs) and NFTs are extensively distributed throughout relay stations in the ascending auditory pathway (Sinha et al., 1993; Parvizi et al., 2001; Rub et al., 2016). Many AD mouse models have been used to explore hearing dysfunction and their underlying mechanisms (summarized in Table 1). Studies have shown that AD mouse models initially exhibit high-frequency hearing loss and finally progress to the entire frequency. 5xFAD and APP/PS1 mice are mainly characterized by β-amyloid plaque deposition and show elevated auditory brainstem response (ABR) thresholds. 5xFAD mice co-express gene mutations in five FAD and can generate Aβ deposits rapidly (Oakley et al., 2006). In 5xFAD mice, amyloid depositions were observed at 2 months of age, while cochlear histopathology revealed a large amount of apical and basal hair cell loss at 13 months of age (O’Leary et al., 2017). The onset of auditory dysfunctions in APP/PS1 mice preceded before neuropathological changes, suggesting that acoustic measurements might be a non-invasive indicator for AD detection (Liu et al., 2020). 3xTg-AD mice express 3 AD-related transgenes, and its neuropathology developments are similar to FAD patients, characterized by Aβ deposition, tau pathology, and neuroinflammation. In 3xTg-AD mice, a reduction of SGN’s relative densities was observed at 9–12 months of age. A transgenic mouse model with overexpression of Aβ peptides in hair cells was established by Omata et al. (2016) and high-frequency hearing impairments were found at 4 months of age. Aligned with the electrophysiological assessment, basal hair cell loss was observed. They further established another model overexpressing tau pathology but found no significant hearing dysfunctions. Nevertheless, double transgenic mice showed an advanced and exaggerated hearing impairments, suggesting that Aβ deposition was a fundamental pathological etiology for hearing defects exhibited by AD and that tau pathology enhanced the dysfunction (Omata et al., 2016). Many previous studies have shown that both oxidative stress and apoptosis play essential roles in the death of hair cells (Yu et al., 2017; Li et al., 2018a), whether hair cell loss observed in the researches can be attributed to Aβ-induced oxidative stress and cell apoptosis need further study.

**TABLE 1:** List of AD, HD, ASD mice models that have been used for auditory function and anatomy study.

	Mice lines	Mutations	Neuropathological abnormalities/manifestations	Auditory dysfunction	Auditory circuit anatomy	Reporter and year of publication
AD	3xTg-AD	*APP*	• Neuroinflammation:	• ABR: thresholds increased at 9-month-old	• SGNs loss: at 9–12 months	Wang and Wu, 2015
		*PSEN1*	6-month –old	• DPOAE: normal		
		*Tau*	• Aβ deposits:			
			Initiate at 6-month-old			
			Apparent at 12-month-old			
			• Tau pathology:			
			12-month-old			
			• Synaptic dysfunction			
	5xFAD	APP K670N/M671L	• Aβ deposition:	• ASR: thresholds elevated at 3–4 months	• HC loss:	O’Leary et al., 2017
		(Swedish) + I716V	Onset at 2-month-old	• ABR: thresholds increased at 8–32 kHz	Apical and basal IHCs	
		(Florida) + V717I (London) PS1 M146L + L286V	Apparent at 4-month-old • Neurodegeneration and cognitive deficits: 4–5 months	at 13–14 months	And OHCs at 15–16 months	
	APP/PS1	*APP*	• Aβ deposition:	• ABR:	/	Liu et al., 2021
		*PSEN1*	6–7 months	1) High frequency increased at 2–3 months; 2) Whole frequency increased at 3–4 months;		
				3) Wave IV and V reduction at 3-month-old • DPOAE 16 and 20 kHz increased at 3-month-old • CM: normal		
HD	Hdh(CAG)^150^	Huntingtin knock-in	• mHtt aggregation: at 10–14 months	• ABR: thresholds at 4 and 8 kHz increased at 15-month-old	• Spiral ganglion/the organ of Corti: 1) mHtt aggregation; 2) Reduced CKB expression; 3) At 15–20 months	Lin et al., 2011b
	R6/2	Huntingtin (around 150 CAG repeats)	• mHtt aggregation: 5–6 weeks of age	• ABR: thresholds increased at 2–3 months • DPOAE: thresholds increased at 2–3 months	• Reduced prestin level: at 3-month-old • HC loss: at 3-month-old	Wang and Wu, 2015
					• SGNs loss: at 3-month-old	
ASD	16p11.2 deletion ^±^	16p11.2 deletion	• Low body weight	• No ASR at any decibel level	/	Yang et al., 2015
			• Perinatal mortality	• No ABR to wide frequencies:		
			• Spontaneous locomotor activity	Between 8 and 100 kHz;		
			• Sporadic motor stereotypies			
	*Cntnap2* ^–/–^	*Cntnap2* knockout	• Reduced social interaction • Hyperactivity • Repetitive behaviors • Reduced ultrasonic vocalization output	• Auditory-processing dissociation: 1) Impairs Silent Gap Detection 2) Enhanced Tone Discrimination	• Medial Geniculate Nucleus: 1) Reduced neuron numbers 2) Smaller neurons	Truong et al., 2015
	*Adnp* ^±^	truncated Adnp	• Irregular tooth eruption • Short stature • Social and vocal impediments • Motor delays • Learning and memory deficits	• ABR: Increased thresholds; Prolonged latency; at 2.5-month-old	• Normal hair-cell morphology at P0 • Expression of autism and auditory related proteins 1) Auditory cortex: Decreased ChAT in male *Adnp*^±^ Decreased PVALB in male *Adnp*^±^ 2) Cerebellum: Increased GAD67 in female *Adnp*^±^ Decreased VGLUT2, CX32, and ChAT in female *Adnp*^±^	Hacohen-Kleiman et al., 2019

*ABR, auditory brainstem responses; *ADNP*, activity-dependent neuroprotective protein; *APP*, amyloid precursor proteins; *ASR*, auditory startle response; CKB, brain-type creatine kinase; *CNTNAP2*, contactin-associated protein-like 2; DPOAE, distortion product otoacoustic emission; HC, hair cell; IHC, inner hair cell; mHtt, mutant Huntingtin; OHC, Outer Hair Cell; *PSEN1*, Presenilin 1; SGNs, Spiral Ganglion Neurons.*

*“/” means that information on the item is not available in the relevant research.*

Clinical literature suggests that midlife hearing loss is independently correlated with accelerated progression of sporadic AD and incident dementia. The degree of hearing loss was positively associated with an increased risk of dementia. AD-related neuropathology was found in the central auditory pathway but was not clinically identified in the peripheral auditory pathway. Although multiple clinical studies have been designed to determine the relationship between hearing loss and AD, there are still some flaws in the experimental design. Most of the important information has been neglected, including the extent of hearing loss, measurements of auditory processing, differences between sexes, and auditory condition in different AD classifications, resulting in restricted access to information to determine the relationship between auditory dysfunction and AD. Mouse model studies further illustrated that hearing loss is associated with AD development. In the APP/PS1 mouse model, the shifts of ABR and distortion product otoacoustic emission (DPOAE) preceded the neuropathy observed in the brain. Loss of hair cells and SGNs was observed in AD mouse models, which was likely induced by the spread of AD-related neuropathology (Aβ deposition and tau pathology) in the cochlea. However, the three AD-related mouse models were all designed with mutations in FAD genes, which could not completely mimic the pathogenesis of sporadic AD. The central auditory pathway has not yet been studied in these mouse models. High-frequency hearing loss has been observed in patients with AD and incident dementia. Moreover, both the central and peripheral auditory pathways are affected by AD-related neuropathology, but the concrete cochlear pathology is still debated. The results vary among studies due to different mouse models, sampling times, and hearing measurements. Hence, for a comprehensive understanding of AD-related hearing loss, standard observation criteria should be established in further studies.

## Huntington’s Disease

Huntington’s disease is an autosomal-dominantly inherited disorder with a mean prevalence of 2.71 per 100,000 individuals worldwide (Pringsheim et al., 2012). HD manifests with midlife cognitive impairment, motor incoordination, and psychiatric symptoms (Martin and Gusella, 1986; Walker, 2007). Late-stage HD patients often present with auditory sensory, processing, and memory problems other than typical dysfunctions. Studies have illustrated that hearing impairment is involved in and is closely correlated with motor deficits in HD (Josiassen et al., 1984; Lin et al., 2011b). Lin et al. (2011b) recruited 19 HD patients and assessed hearing impairments using PTA and ABR. The PTA thresholds showed that an average increase of 15 dB was detected in high frequencies of HD patients, and no significant differences were observed in latency and inter-peak intervals of ABRs, indicating that hearing impairments in HD were more associated with the peripheral auditory pathway than retrocochlear lesions (Lin et al., 2011b). In contrast, other researchers found that HD patients displayed normal sound sensation, but with a significant decrease in speech understanding and sound source lateralization, suggesting that HD-associated neuropathology affects the central auditory system (cortical and subcortical parts) (Beste et al., 2008; Saft et al., 2008; Profant et al., 2017). Wetter et al. (2005) revealed that HD patients had delayed auditory event-related potentials (ERPs), which were also found in individuals at risk for HD. These findings showed HD-related dysfunction during sound processing (Homberg et al., 1986; Josiassen et al., 1988; Wetter et al., 2005).

**FIGURE 2:**
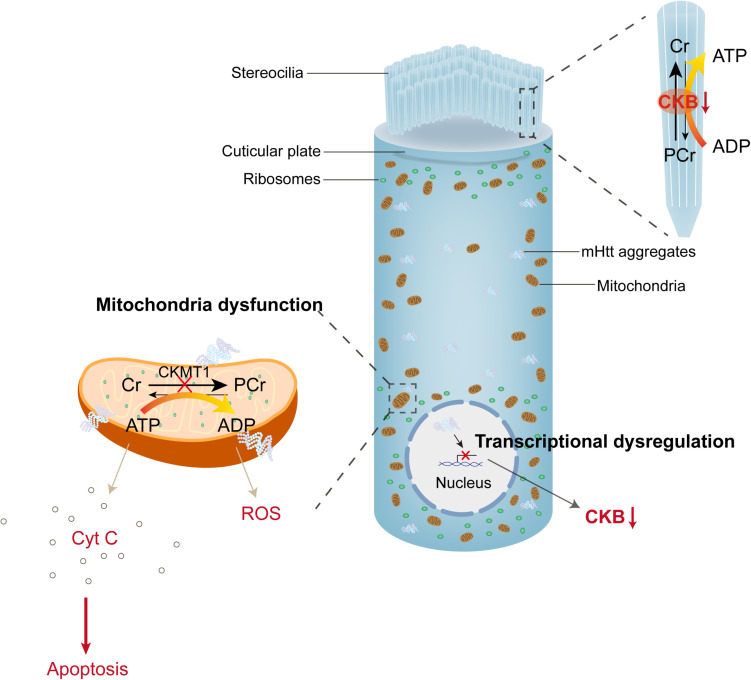
mHtt aggregates induced toxicity and dysregulated PCr-CK system in hair cells. The PCr-CK system plays a critical role in providing ATP in hair bundles of hair cells, mitochondrial creatine kinase (CKMT1) phosphorylates creatine (Cr) to phosphocreatine (PCr). In the stereocilia, brain-type creatine kinase (CKB) regenerates ATP from PCr. Expression of mHtt in hair cells impairs the function of mitochondria, releases cytochrome C and reactive oxygen species (ROS) to the cytoplasm. On the other hand, mHtt aggregates lead to protein sequestration (including many transcriptional factors), then induce transcriptional dysregulation, which reduces the expression of CKB (creatine kinase).

The pathophysiological mechanisms underlying HD-related auditory dysfunction are poorly understood; nonetheless, recent studies in transgenic mouse models provide new insight into these mechanisms (Walker, 2007). Aggregated mutant huntingtin (mHtt) is the most classic cellular pathological characteristic of HD; extra amplificated CAG repeats in exon 1 of *huntingtin* lead to polyglutamine (polyQ) extension at the N-terminal of Htt protein, and mutant Htt accumulates to cause neuronal loss (Figure 2; Macdonald et al., 1993; Mangiarini et al., 1996; Ha and Fung, 2012). Neuronal loss preferentially affects the cortico-striatal circuits, which leads to characteristic chorea, and as HD progresses, mHtt spreads to peripheral tissues, including the inner ear (Vonsattel and DiFiglia, 1998; Cepeda et al., 2007; Snowden, 2017). Mouse models were also used to illustrate auditory dysfunction and pathology in HD (summarized in Table 1). R6/2-HD mice express mHtt and present with HD-related phenotypes at 5–6 weeks of age. Lin et al. (2011b) found that R6/2-HD mice exhibited approximately 10 dB elevation of ABR thresholds at 2 months of age before the presentation of motor deficits. After 3 weeks, the motor defects became apparent, and a 30 dB threshold shift for click stimuli and a 15 dB threshold shift for tone bursts at all frequencies were also observed. In addition, they found no difference in ABR latency and peak intervals between R6/2-HD mice and wild type mice (Lin et al., 2011b). As described in Wang’s research, R6/2-HD mice exhibited increased distortion product otoacoustic emission and ABR thresholds at 2–3 months of age. Furthermore, the relative expression of prestin was reduced in OHCs, which was reported to be responsible for dysfunction in hearing sensitivity and frequency selectivity. Cochlear SGN reduction and hair cell loss (especially OHCs) were observed histologically after that (Wang and Wu, 2015), suggesting that mHtt pathology in the central and peripheral auditory system contributed to the presence of hearing impairments in HD. Hdh(CAG)^150^ mice are knock-in mice that carry 150 CAG repeats on the Htt locus, of which mHtt aggregation in the nervous system and HD-related characteristics initiate at approximately 10 months of age. In Hdh(CAG)^150^ mice, thresholds measured by click and tone bursts ABR analysis at 15 months of age revealed that approximately 20 dB thresholds were increased for click and tone bursts at frequencies of 4 and 8 kHz. No differences were observed at frequencies of 16 and 32 kHz because wild type mice developed presbycusis at 15 months of age (Lin et al., 2011b). Moreover, aggregated mHtt and continuing loss of brain-type creatine kinase (CKB), which was previously reported to decline in HD patients, were both obtained in the organ of Corti and the spiral ganglion in both mouse models (Perluigi et al., 2005; Sorolla et al., 2008; Kim et al., 2010; Lin et al., 2011b). Aggregation of mHtt is thought to affect many transcriptional factors and induce mitochondrial dysfunction, which directly leads to the release of cytochrome C and oxidative stress (Kim and Kim, 2014). CKB, a cytosolic enzyme, can regenerate ATP by reversibly transferring high-energy phosphate from phosphocreatine (PCr) to ADP (Jacobus and Lehninger, 1973; Wallimann et al., 1992; Wyss and Kaddurah-Daouk, 2000). CKB also localizes in cochlear hair cells and ligaments and is critical for hearing function (Spicer and Schulte, 1992; Spicer et al., 1997). CKB-knockout mice presented with high-tone hearing loss that can be restored by dietary creatine supplements (Shin et al., 2007; Lin et al., 2011c). These studies suggest that CKB dysregulation may be associated with HD-related auditory dysfunction, synergistic with mHtt (Figure 2).

Auditory dysfunction appears to be authentic for HD. Clinical studies have shown that hearing impairments and auditory processing dysfunction are present in HD patients. Delayed ERPs are suggested to be a potential predictor of HD. While there is no consensus that auditory sense, processing, or discrimination is uniparted or jointly present in HD, more research objectives, detailed acoustic measurements, and specified auditory items should be included. Hearing impairment is solid in HD mouse models, and hearing loss precedes the occurrence of motor defects and worsens with the progression of HD in R6/2-HD mice. The loss of hair cells and SGNs was also observed. Hdh(CAG)^150^ mice exhibited significant low-frequency hearing impairment compared to wild type mice, which was accompanied by presbycusis-related high-frequency hearing loss, suggesting that hearing impairments in HD patients was not merely related to the auditory pathway degeneration caused by natural aging and clarifying that hearing loss was authentic to HD, while the cochlear anatomy has not been assessed in Hdh(CAG)^150^ mice. Moreover, pathological studies of the central auditory pathway, including cochlear nuclei, superior olivary complex, and auditory cortex, should be performed to determine auditory-related lesions and guide auditory-related tests carried out for HD patients and high-risk groups.

## Parkinson’s Disease

Parkinson’s disease is a neurodegenerative disorder characterized by static tremor, bradykinesia, rigidity, and postural instability, affecting 1–2 per 1,000 individuals (Dorsey et al., 2007; Ascherio and Schwarzschild, 2016). Before the onset of typical motor symptoms, patients with PD often manifest with cognitive impairment, olfactory dysfunction, fatigue, etc. (Postuma et al., 2012; Khoo et al., 2013). Recently, hearing impairment has been considered as another non-motor feature in PD patients. Studies have shown that the incidence of developing PD in the hearing loss group was 1.77 higher than in the control group (Lai et al., 2014b), and high-frequency hearing impairment was observed in PD patients without self-perceiving (Yylmaz et al., 2009; Santos-Garcia et al., 2010; Shetty et al., 2019), which is positively related to PD duration and worsens as it progresses (Scarpa et al., 2020). PTA results showed an average elevation of 10 dB in 4 and 8 kHz in PD patients, and significantly increased latencies in wave V and interpeak were obtained (Yylmaz et al., 2009). PTA performed among the relatively younger (age < 55 years old) population of PD showed that thresholds were elevated at high frequency and low to mid frequencies. This ratio was even higher for low-mid-frequency hearing loss. Concurrently, the brain stem auditory-evoked potentials were comparable to the control group, indicating that hearing loss in PD was independent of aging and that the underlying mechanism appeared to be peripheral according to the study (Shetty et al., 2019). Vitale et al. (2013) calculated the proportion of different degrees of hearing loss in 75 patients with PD and found that 89% of them had mild to moderate hearing loss, and 11% had severe hearing loss. In addition, they revealed that the prevalence of PD with hearing impairments was higher in the male elderly (Vitale et al., 2013). Whole frequencies of distortion product otoacoustic emission thresholds in PD patients also increased. It can be alleviated by dopaminergic treatment (Georgiev et al., 2015; Pisani et al., 2015), which uncovered an undermined dopamine-dependent cochlear dysfunction undermined. Sisto et al. (2020) found that the ipsilateral cochlear dysfunction developed in parallel with asymmetric motor impairment. The abilities of speech discrimination and sound lateralization were also markedly reduced in PD patients (Lewald et al., 2004; Vitale et al., 2016; Folmer et al., 2017), and abnormal auditory evoked potentials were suggested as a measurement of PD duration and severity (Yylmaz et al., 2009; Jafari et al., 2020).

The association between hearing dysfunction and PD suggests a common neuropathological background. Lewy pathology and dopaminergic neuronal degeneration are two primary neuropathological features of PD that spread as PD progresses (Dickson et al., 2009; Dickson, 2012; Kordower et al., 2013). Other protein aggregations, such as Aβ plaques and NFTs, are also present in the nervous system of PD patients (Kalia and Lang, 2015). Lewy pathology consists of insoluble misfolded α-synuclein that can be found in certain regions of the central and peripheral nervous system in PD (Wakabayashi et al., 1989; Spillantini et al., 1997; Beach et al., 2010; Del Tredici et al., 2010; Goedert et al., 2013). In the inner ear, α-synuclein is located predominantly in the efferent neuronal system, especially in the OHC, and contributes to the physiological maintenance of auditory function. Hence, Lewy pathology in the auditory system has been speculated to be associated with PD-related auditory disorders (Akil et al., 2008; Park et al., 2011). On the other hand, common neurotransmitters between the auditory system and basal ganglia were indicated by the curative effect of dopaminergic therapy on auditory responses (Rey et al., 1996; Erro et al., 2015; Georgiev et al., 2015; Pisani et al., 2015). Furthermore, dopamine and glutamate mediate the synaptic interplay oppositely in the basal ganglia. In the auditory system, dopamine also counteracts the excitotoxic effects caused by glutamate to modulate auditory processing and neural plasticity. Since glutamate overdose can induce excitotoxic damage to primary auditory neurons, it was speculated that excessive glutamate caused by the degeneration of dopaminergic neurons might account for PD-related auditory dysfunction (Lendvai et al., 2011). Other common underlying mechanisms, including mitochondrial dysfunction, reduced neurotransmitter levels, perturbed protein homeostasis, and oxidative stress, have also been discussed in previous studies (Simon and Johns, 1999; Raza et al., 2019).

There have been no reports on auditory dysfunction and auditory anatomy in PD mouse model. Although manipulation of specific genes reported in familial PD, including transgenic overexpression for α-synuclein and leucine-rich repeat kinase 2 and knockout models for Parkin, DJ-1, phosphatase, and tensin homolog-induced novel kinase 1, made it possible to establish many mouse models, none of them recapitulate key clinical and neuropathological features of PD entirely, especially in the absence of neurodegeneration of dopaminergic neurons (Dawson et al., 2010). While the objective is to gain insight into the molecular mechanisms underlying auditory dysfunction and PD, studies of auditory function in mouse models with specific gene mutations are still needed. PD is a global neurodegenerative disorder that affects the central and peripheral nervous system, and extensive literature indicated a broad range of auditory dysfunctions from the peripheral auditory system to cortical areas in PD (Pekkonen et al., 1995; Kofler et al., 2001; Putzki et al., 2008; Bronnick et al., 2010; Pisani et al., 2015; Potter-Nerger et al., 2015; Seidel et al., 2015; Liu et al., 2017; Shalash et al., 2017; Guducu et al., 2019), asymptomatic hearing impairments appeared to be a newly non-motor manifestation of both early and late-onset PD, and it can be speculated that the natural aging process combined with PD-related neurodegenerative changes coalesce to induce that. Moreover, the central auditory dysfunctions, including abnormal speech discrimination and sound lateralization, cannot be ignored. The literature suggests hearing measurement as a non-invasive potential biomarker and indicator of disease severity for PD, widespread alpha-synuclein neuropathology, and loss of dopaminergic neurons were suspected of interfering with such auditory dysfunction, and PD mouse models should be applied for precise assessment of hearing function and pathological mechanism exploration.

## Autism Spectrum Disorder

Autism spectrum disorder is a neurodevelopmental disorder characterized by social isolation, stereotypical behaviors, and interests, with a prevalence of approximately 1% worldwide and has a strong male predominance (Johnson et al., 2007; Lai et al., 2014a). Sense dysfunction, including the feeling of touch, smell, taste, vision, and hearing, is another feature of ASD. The pathogenesis of ASD is not entirely understood, but comorbidities and maternal exposures in placental life may act as risk factors (Arndt et al., 2005). Researchers have suggested that genetic polymorphisms and environmental factors jointly contribute to the phenotypic variation in ASD (Bailey et al., 1995; Veenstra-VanderWeele et al., 2004; Lopez-Rangel and Lewis, 2006). Cerebellar and brainstem hypoplasia was observed in patients with ASD (Courchesne et al., 1988; Hashimoto et al., 1992, 1995), and multiregional neuropathy defects have been identified, including a reduced number of Purkinje cells in the cerebellum, delayed neuron maturation of the forebrain, abnormal development of the frontal and temporal lobes, and sporadic malformation in the brainstem and neocortex (Hardan et al., 2004; Pickett and London, 2005; Lainhart, 2006; Wegiel et al., 2010, 2014; Hampson and Blatt, 2015). These structural abnormalities lead to typical behavioral manifestations and sense dysfunctions in ASD.

Currently, most previous studies identified increased rates of audiological dysfunctions in ASD, including hearing impairments, hyperacusis, difficulty in sound discrimination with background noise and speech sounds encoding (Tomchek and Dunn, 2007; Russo et al., 2009; Stiegler and Davis, 2010). A higher incidence of hearing loss (from unilateral to bilateral) and hyperacusis was demonstrated in the ASD population (Rosenhall et al., 1999; Demopoulos and Lewine, 2016; Do et al., 2017). Fitzpatrick et al. (2014) found that approximately 29.4% of children with ASD had profound hearing loss and that those children with hearing loss benefited from the use of hearing aids. In addition, hearing dysfunction was attributed to ASD-related neuronal degeneration of the auditory pathway (Smith et al., 2019). In contrast, Szymanski et al. (2012) found a high prevalence of ASD among children with hearing loss, supporting that peripheral auditory dysfunction may be associate with functional impairment in ASD (Demopoulos and Lewine, 2016). Previous studies have provided an abundance of evidence supporting both abnormal structure and function in the auditory brainstem of ASD, but there remains a battery of literature showing that the peripheral auditory manifestations of children with ASD were comparable to controls (Gravel et al., 2006; Tharpe et al., 2006). Beers et al. (2014) reviewed 22 articles about peripheral hearing loss in ASD and concluded that there was no solid evidence for an increased risk of peripheral hearing loss among children with ASD. Tas et al. (2007) also evaluated the auditory function of children with ASD through transient evoked otoacoustic emission and ABR. The positive emission and normal hearing level at ABR revealed an insusceptible peripheral auditory system in patients with ASD. Nevertheless, the ABR results showed a prolonged III–V interpeak latencies (IPLs) in children with autism (Rosenhall et al., 2003; Tas et al., 2007).

Approximately 10% of ASD cases have an identifiable genetic background. Many ASD-related genetic and chromosomal disorders have been shown to present with auditory dysfunction (Table 2), underlying a potential common genetic etiology between ASD and auditory dysfunction. Many chromosomal disorders have been reported to manifest with auditory dysfunctions, ASD, developmental retardation, seizures, facial dysmorphism, and multisystem defects. Deletions and duplications range from specific loci to large segments and comprise a considerable number of related genes. Genes with remarkably high risk accounting for these manifestations are listed in Table 2. Most of them are involved in neuron and synaptic development (Smith et al., 2002; Sinajon et al., 2015; Yang et al., 2015; Lahbib et al., 2019; Wu et al., 2020a), among which only two genes are known to be auditory-related: *ELMOD3* and *FGF2*. *ELMOD3* is involved in autosomal recessive non-syndromic deafness disability (Lahbib et al., 2019), and *FGF2* plays a role in the proliferation and survival of auditory neuroblasts (Wu et al., 2020a). Three monogenic disorders were reported to present with auditory dysfunction and ASD simultaneously, including Fragile X syndrome, *MEIS2* syndrome, and *ADNP* syndrome (Rotschafer et al., 2015; Douglas et al., 2018; Hacohen-Kleiman et al., 2019), related genes all function in brain development, and *MEIS2* is responsible for the development of the inner ear in chicken (Douglas et al., 2018). Restricted information about genes and their functions is insufficient to illustrate the genetic association between auditory dysfunction and ASD. Whether these certified ASD-related genes also participate in auditory function is unclear.

**TABLE 2:** List of ASD-related chromosomal and monogenic disorders that have been reported co-presented with auditory dysfunction.

	Chromosomal/genetical abnormalities	Map position	Incidence	Manifestations	Potential related genes and function	References
**Chromosomal disorder**	chromosomal 13q12→q13 deletion	• deletion at the distal third of band 13q12 • deletion at the proximal two-thirds of band 13q13	/	• auditory processing defects • autism spectrum disorder • language deficit	• *NBEA*: 1) encodes a neuron-specific multidomain protein 2) functions as a protein kinase anchor protein 3) post-Golgi neuronal membrane trafficking • *MAB21L1*: neural development • *DCAMKL1*: 1) encodes a brain-specific transmembrane kinase 2) cortical development • *DCX*: 1) encodes doublecortin, a brain-specific putative signaling protein 2) neuronal migration • *MADH9*: a member of the *SMAD* family 1) mediate the TGF beta signaling pathway 2) proliferation and differentiation of many different cell types 3) synaptic junction differentiation	Smith et al., 2002
	16p11.2 deletions and duplications	heterozygous deletions and duplications of 16p11.2	1% of individuals with autism	• auditory dysfunction: 1) hearing loss 2) absence of acoustic startle responses • autism spectrum disorder • developmental delays, speech delay • obesity (deletion) and low body weight (duplication) • intellectual impairment • psychiatric disorders • seizures, syringomyelia • cardiac defects • motor hypotonia • immune deficiency	• *KCTD13*: • encodes the polymerase delta-interacting protein 1 (PDIP1) • regulation of cell cycle during neurogenesis • *SEZ6L2*: epilepsy and language disorders • *MAPK3*: 1) a member of the MAP kinase family 2) cellular proliferation, differentiation, and cell cycle • *NRX1*, *NRXN3:* synaptic transmission and cell-cell interaction • *CHD8*, *EHMT1, MECP2, SOX5, TBF4, SATB2, FOXP1*: chromatin modifiers and transcription factors • *FMR1* and *CEP290*: intellectual disability	Yang et al., 2015
	chromosome 8q22.2-q22.3 deletion	deletion at chromosome 8q22.2-q22.3	/	• bilateral hearing loss: hypoplastic auditory canals • autism spectrum disorder • macrocephaly • childhood seizure disorder • moderate intellectual disability • facial phenotype • congenital heart defect	• *GRHL2*: non-syndromic autosomal dominant deafness gene • *VPS13B*: the causative gene for Cohen syndrome • *SPAG1*: responsible for primary ciliary dyskinesia • *RRM2B*: encodes a small subunit of p53 mitochondrial DNA disorders and depletions • *NCALD*: neuronal signal transduction process	Sinajon et al., 2015
	chromosome 2p11.2 deletion	homozygous deletion in 2p11.2	/	• hearing impairment • autism spectrum disorder • intellectual disability • language delay • behavioral disturbances	• *ELMOD3*: involves in autosomal recessive non-syndromic deafness-88 (DFNB88) • *CAPG*: 1) member of actin regulatory proteins 2) cytoskeletal rearrangements regulation 3) involves in Rett syndrome • *SH2D6*: signal transduction of receptor tyrosine kinase pathways	Lahbib et al., 2019
	Chromosome 4q deletion and 7q duplication	• deletion of chromosome 4 • microduplication of chromosome 7	/	• unilateral hearing impairment • autism spectrum disorder • multisystem malformation: 1) facial dysmorphism: microcephaly 2) ocular malformation: ocular hypertelorism; exophthalmos 3) auditory malformation: low-set ears 4) appendicular malformation:	• *SPATA5*: 1) mitochondrial function (morphology and dynamics) 2) neuronal development 3) spermatogenesis • *FGF2*: 6) Angiogenesis 6) cell survival, division, differentiation, and migration 6) proliferation and survival of auditory neuroblast	Wu et al., 2020a
				single palmar flexion crease; overlapping toes 6) cardiopulmonary system: discontinued cyanosis recurrent respiratory infections patent foramen ovale tracheobronchomalacia 6) nervous system: persistent falcine sinus with a thin corpus callosum	• limb development • wound healing • tumor growth • *NAA15*: encodes a component of the Nat A Nacetyl-transferase complex, which tethering the complex to the ribosome for posttranslational modification of proteins • *SMAD1*: development of pulmonary hypertension • *HHIP*: development of lung malformation	
**Monogenic disorder**	Fragile X Syndrome	• *FMR1* gene locates in Xq27.3 • *FMR1* gene silencing by: • amplification of a CGG repeat • methylation of the promoter region	1 in 1250 males and 1 in 2500 females	• hearing loss: 1) elevated cortical responses to sound stimuli 2) aberrant ABRs • autism spectrum disorder • cognitive impairments • seizures • aberrant dendritic spine morphology • enhancement of response to sensory stimuli	• a modulator of mRNA translation • regulates synaptic proteins production	Rotschafer et al., 2015
	*MEIS2*(MRG1)	locates in chromosome 15q14	/	• hearing loss • autism spectrum disorder	• encodes a homeodomain protein implicated as a transcriptional activator	Douglas et al., 2018
				• atrial or ventricular septal defect	• cell proliferation	
				• developmental delay	• development of inner ear in chickens	
				• intellectual disability	• development of heart, brain, limb	
				• short stature	• differentiation of various tissues and organs	
				• cleft palate		
				• gastrointestinal, skeletal, limb, and skin abnormalities		
	*ADNP* syndrome	locates in chromosome 20	0.17% of individuals with autism	• mild hearing loss: > 10% of children • autism spectrum disorder	• regulates ion channels genes • regulates the protein translation process	Hacohen-Kleiman et al., 2019
				• intellectual, motor, social, and speech delays/disabilities	• neural tube closure • associates with the cytoskeleton	
					• synaptic plasticity • microtubule-dependent axonal transport	
					• dendritic spine formation	
					• brain development	
					• mental function	

**CAPG*, Capping Actin Protein, Gelsolin Like; *CEP290*, Centrosomal Protein 290; *CHD8*: Chromodomain Helicase Dna Binding Protein 8; *DCAMKL1*, Doublecortin Like Kinase 1; *DCX*, Doublecortin; *EHMT1*, Euchromatic Histone Lysine Methyltransferase 1; *ELMOD3*, Domain Containing 3; *FGF2*, Fibroblast Growth Factor-2; *FMR1*, Fmrp Translational Regulator 1; *FOXP1*, Forkhead Box P1; *GRHL2*, Grainyhead Like Transcription Factor 2; *HHIP*, Hedgehog Interacting Protein; *KCTD1*3, Potassium Channel Tetramerization Domain Containing 13; *MAB21L*, Mab-21 Like 1; *MADH*9, Smad Family Member 9; *MAPK*3, Mitogen-Activated Protein Kinase 3; *MECP2*, Methyl-Cpg Binding Protein 2; *NAA15*, N-Alpha-Acetyltransferase 15; *NBEA*, Neurobeachin; *NCALD*, Neurocalcin De*lta; NRX1*, nucleoredoxin 1; *NRXN*3, neurexin 3; *RRM2B*, ribonucleotide reductase regulatory TP53 inducible subunit M2B; *SATB2*, SATB homeobox 2; *SEZ6L2*, seizure related 6 homolog like *2; SH2D6*, SH2 domain containing 6; *SMAD1*, SMAD family member 1; *SOX5*, SRY-box transcription factor 5; *SPAG*1, sperm associated antigen 1; *SPATA5*, spermatogenesis associated 5; *VPS13B*, vacuolar protein *sorting 13* homolog.*

*“/” means that information on the item is not available in the relevant research.*

ASD-related mouse models have been developed to study auditory dysfunction (Table 1). Chromosomal disorder mice characterized by 16p 11.2 deletions showed whole frequency increased ABR and auditory startle response (ASR) thresholds, indicating that genes located in the area were responsible for auditory dysfunctions, of which *KCTD13, SEZ6L2*, and *MAPK3* were considered to be highly correlated with autism (Konyukh et al., 2011; Golzio et al., 2012; Blumenthal et al., 2014; Yang et al., 2015), while their relationship with the auditory function has not been determined. Monogenic disorder mice were also studied; *Fmr1*^–/–^, *Cntnap2*^–/–^ and *Adnp*^±^ mice presented with classical characteristics of ASD and showed impaired hearing and auditory process functions. Anatomy of auditory circuits, such as the ventral cochlear nucleus and the medial nucleus of the trapezoid body exhibited reduced neuron size and number. Altered hearing-related protein levels, including VGAT, ChAT, and GAD67, were observed in the auditory cortex and cerebellum (Rotschafer et al., 2015; Ruby et al., 2015; Truong et al., 2015; Hacohen-Kleiman et al., 2019), underlying a central auditory and synaptic pathology.

Increased rates of auditory dysfunction, including hearing impairments, hyperacusis, difficulties in sound discrimination, and speech sounds encoding, were detected in patients with ASD. ASD children with hearing impairments were identified later than those with normal hearing for auditory disorders and related communication delays. Although hearing impairment is an uncommon manifestation of ASD, both diseases affect communication abilities in children, and hearing impairment may contribute to the development of ASD. Comprehensive audiological assessments of confirmed and suspected ASD in children and early hearing interventions are recommended to improve social communication and reduce the aggression of ASD. ASD with chromosomal and monogenetic disorders has been shown to manifest with hearing impairments and auditory process problems, which correspond to auditory dysfunctions in ASD mouse models, and reduced neuron size and number were observed in auditory brainstem nuclei. Studies have also reviewed the genes that might be involved in chromosomal and monogenetic disorders. They concluded that most of them function in neuronal development, suggesting that defective neuropathy in the auditory pathway leads to hearing dysfunction and raises the idea that ASD-related genes may act as potential deafness genes. Hence, specific transgenic mouse models should be applied to clarify their function and influence on the auditory system. For further analysis, next-generation sequencing should be applied to identify more potential ASD-related candidate genes for deafness.

## Conclusion and Perspectives

In this review, we presented research on hearing loss in four common neurological disorders (AD, PD, HD, and ASD) and concluded that hearing loss was present in these four disorders. However, the related auditory lesions and underlying mechanisms vary among them.

In AD, high-frequency hearing loss was observed in both the patient and mouse models. Moreover, Aβ deposition appeared to be the initial neurological etiology. Auditory studies on AD mouse models raise the possibility that the auditory pathway is more sensitive to AD-related neuropathology and auditory dysfunction, especially hearing loss, presents before the onset of cognitive impairments. Thus, auditory measurements can provide a reference for preliminary diagnosis and early interventions for patients with AD. Hearing impairments and auditory processing dysfunction have been observed in HD patients. In R6/2-HD mice, hearing loss precedes the characterized presentations of HD, and in the SGNs of Hdh(CAG)^150^ mice, mHtt aggregation was observed. However, for a comprehensive understanding of auditory dysfunction in AD and HD, more clinical trials involving more subjects and including a variety of detailed auditory measurements should be carried out, and complete studies on auditory circuitry (from the cochlea to the auditory cortex) of mouse models should be conducted in the future.

A broad range of auditory dysfunctions, including hearing loss, abnormal speech discrimination, and sound lateralization, have been reported in PD, and asymptomatic hearing impairments appear to be a new non-motor symptom of PD patients, and hearing measurements may act as a non-invasive potential biomarker and indicator of disease severity. There are no transgenic mouse models that can completely mimic the important neuropathological features of PD, especially the neurodegeneration of dopaminergic neurons. Hence, better PD mouse models should be established, and to gain insight into the underlying molecular mechanisms, auditory studies in existing mouse models still worth exploring.

Many auditory dysfunctions, including hearing impairments, hyperacusis, difficulty in sound discrimination, and speech sound encoding, have been detected in patients with ASD. Concomitant hearing loss makes the diagnosis of ASD more challenging. As both disorders affect communication abilities in children and early hearing interventions have been reported to improve social communication and reduce aggression in ASD, comprehensive audiological assessments should be carried out in confirmed and suspected ASD in children. Moreover, approximately 10% of ASD cases have an identifiable genetic background. Clinical and transgenic mouse model studies revealed the involvement of hearing impairments, raising the possibility that associated genes may act as potential deafness genes. Hence, more potential ASD-related candidate genes should be identified, and specific transgenic mouse models should be applied to explore the function of autism-related genes in the auditory system.

Sensorineural hearing loss affects a large population of people worldwide, and the impact of hearing loss is broad and profound, including delayed language development in children, social isolation, and psychological illness. Hearing loss is not only present in neurological disorders mentioned above but can also affect the prognosis of these diseases to some extent. Hence, exploring hearing loss in neurological disorders is beneficial for understanding the pathogenesis and improving the prognosis of these diseases.

## Author Contributions

SL and CC wrote the manuscript. LL, XM, XZ, and AL collated the resource. JC, XQ, and XG wrote and reviewed the manuscript. All authors contributed to the article and approved the submitted version.

## Conflict of Interest

The authors declare that the research was conducted in the absence of any commercial or financial relationships that could be construed as a potential conflict of interest.

## Publisher’s Note

All claims expressed in this article are solely those of the authors and do not necessarily represent those of their affiliated organizations, or those of the publisher, the editors and the reviewers. Any product that may be evaluated in this article, or claim that may be made by its manufacturer, is not guaranteed or endorsed by the publisher.

## Footnotes

1
https://www.who.int
